# Myocardial native T1 relaxation times are highly dependent on the blood T1 values

**DOI:** 10.1186/1532-429X-17-S1-Q3

**Published:** 2015-02-03

**Authors:** Jesus G Mirelis, Javier Sanchez-Gonzalez, Esther Gonzalez-Lopez, Josebe Goirigolzarri-Artaza, Maria Gallego-Delgado, Ana Garcia-Alvarez, Jose Manuel Garcia-Ruiz, Leticia Fernandez-Friera, Rodrigo Fernandez-Jimenez, Gonzalo Lopez-Martin, Javier Sanz, Valentin Fuster, Borja Ibanez

**Affiliations:** 1 Cardiology, Puerta de Hierro, Madrid, Spain; 2 CNIC, Madrid, Spain; 3 Cardiology, Hospital Clinic, Barcelona, Spain; 4 Philips Healthcare, Madrid, Spain; 5 The Zena and Michael A. Wiener Cardiovascular Institute, Mount Sinai School of Medicine., New York, NY, USA; 6 Hospital Clinico San Carlos, Madrid, Spain

## Background

Pre- (native) and post-contrast myocardial T1 time calculated from T1 mapping sequences on Magnetic Resonance (MR) is currently being applied to study diffuse myocardial fibrosis in various cardiac diseases both in research and clinical area. However, because myocardium is highly perfused, its T1 time may be influenced by that of blood. Our aim was to study this association in an animal model of left ventricular hypertrophy.

## Methods

23 "large white" pigs underwent contrast-enhanced 3.0 T MR (Achieva®, Philips Healthcare) before and several times (up to 5) after surgical ascending aortic banding. T1 mapping was performed using MOLLI before and 40 minutes after administration of Gd-DTPA (0.1 mmol/kg bodyweight; Magnevist, Bayer Healthcare) and a continuous perfusion of Gd-DTPA (0.011mmol/kg/min), so-called equilibrium protocol. T1 maps were generated by pixel-wise fitting of the appropriate model curves to the signal intensities. Signal intensities in pre-and post-contrast images were determined in myocardium, skeletal muscle, fat and blood.

## Results

**Figure 1:**
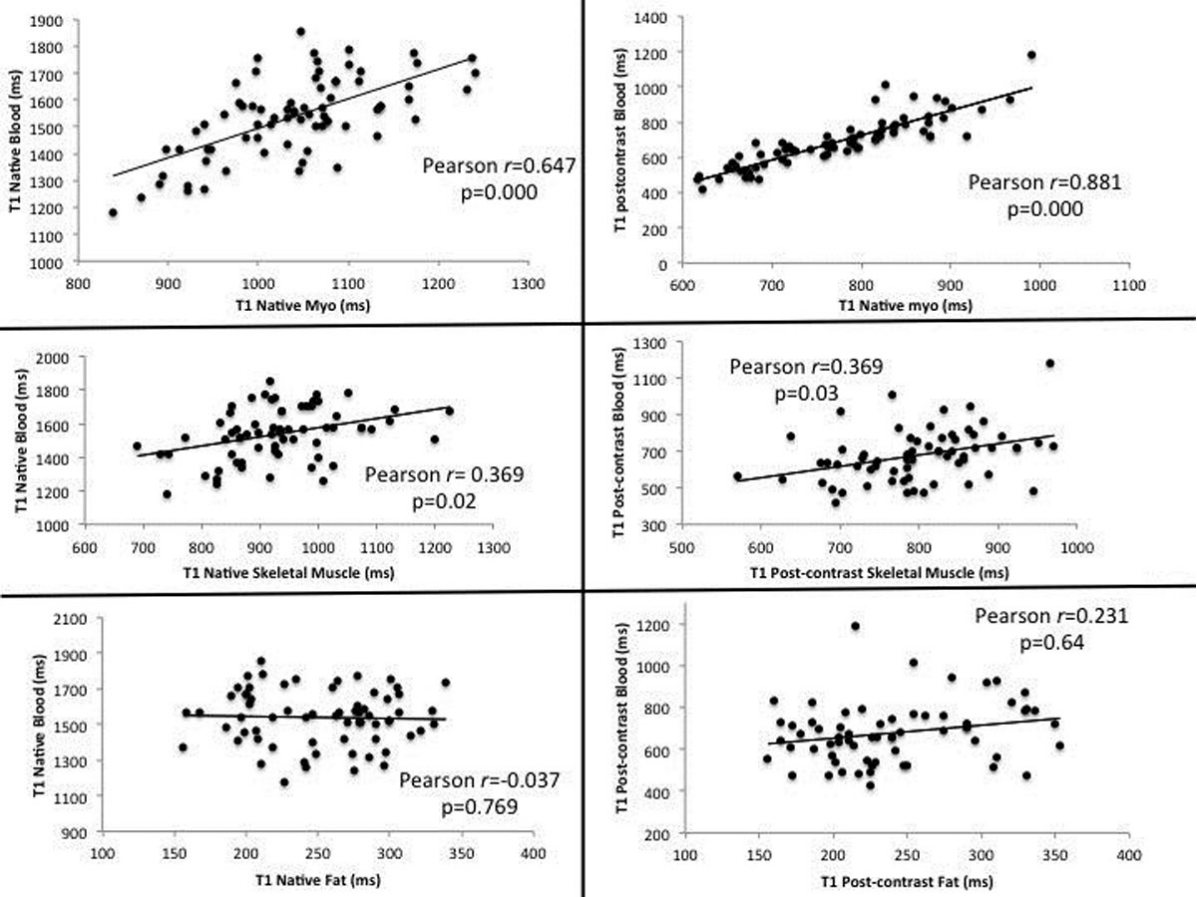
Scatter plots and correlations between blood T1 and different tissues pre- and post-contrast.

Main findings, summarized in Table 1 and Figure 1, were: 1. A strong correlation between myocardial and blood T1 times, which was stronger in the post-contrast setting (Person r=0.65 and r=0.88, respectively; both p<0.01. 2. A weak but significant correlation between skeletal muscle and blood pre and post-contrast T1 times (Person r= 0.37 and p<0.05 for both) relation. 3. Finally, no significant relation was found between fat and blood.

**Table 1:** T1 signal relation between blood and different tissues

Tissue	Level of perfusion	T1 relation between tissues and blood	Person r pre-contrast	Person r post-contrast	p
Myocardium	High	High	0.647	0.881	<0.01 for both

Skeletal muscle	Intermediate	Intermediate	0.369	0.369	<0.05 for both

Fat	Low	Low	-0.037	0.231	>0.05 for both

p: p-value

## Conclusions

Myocardial T1 time appears to be highly influenced by T1 time of the blood. This influence was reduced or nonexistent in tissues with in tissues with intermediate or poor levels of perfusion, such as skeletal muscle or fat, respectively. These findings should be taking into account when interpreting myocardial T1 and its relation with interstitial fibrosis.

## Funding

N/A.

